# RNA-seq transcriptomic profiling of crassulacean acid metabolism pathway in *Dendrobium catenatum*

**DOI:** 10.1038/sdata.2018.252

**Published:** 2018-11-13

**Authors:** Long-Hai Zou, Xiao Wan, Hua Deng, Bao-Qiang Zheng, Bai-Jun Li, Yan Wang

**Affiliations:** 1 Key Laboratory of Tree Breeding and Cultivation of State Forestry Administration; State Key Laboratory of Tree Genetics and Breeding; Research Institute of Forestry, Chinese Academy of Forestry, Beijing 100091, China; 2 Research Institute of Forestry Policy and Information, Chinese Academy of Forestry, Beijing 100091, China

**Keywords:** Plant physiology, Transcriptomics, Drought, RNA sequencing

## Abstract

The regulation of crassulacean acid metabolism (CAM) pathway has recently become a topic of intensive research and has been explored in terms of several aspects, including phylogenetics, genomics, and transcriptomics. Orchidaceae, which contains approximately 9,000 CAM species, is one of the largest lineages using this special photosynthetic pathway. However, no comprehensive transcriptomic profiling focused on CAM regulation in orchid species had previously been performed. In this report, we present two Illumina RNA-seq datasets, including a total of 24 mature leaf samples with 844.4 million reads, from *Dendrobium catenatum* (Orchidaceae), a facultative CAM species. The first dataset was generated from a time-course experiment based on the typical CAM phases in a diel. The second was derived from an experiment on drought stress and stress removal. A series of quality assessments were conducted to verify the reliability of the datasets. These transcriptomic profiling datasets will be useful to explore and understand the essence of CAM regulation.

## Background & Summary

Crassulacean acid metabolism (CAM) is the most important photosynthetic physiology by which plants adapt to seasonal water-limiting areas. A CAM plant can assimilate CO_2_ into the opening stoma during the night and employ it in the Calvin cycle under stoma closure during the following day. This physiological process reduces evaporative demand by performing stomatal opening during the cooler nighttime instead of during the daytime when the transpiration rate would be higher. Hence, plants engaged in CAM have higher water use efficiency than their C_3_ and C_4_ counterparts^1^ and are considered to be important contributors to agriculture in semiarid and arid regions^2–4^.

Although the core CAM pathway has been delineated in detail^5^, the regulatory mechanisms of these and other associated processes, including stomatal movement, carbohydrate metabolism, and transmembrane transport under circadian rhythms, remain largely unknown. The bulk of recent studies in this field have mainly relied on gene phylogenetic analyses^6–8^, genomics^9–11^, proteomics^12,13^, and transcriptomics^13–19^. These published transcriptomic data include species from the genera *Anans* (Bromeliaceae), *Agave* (Agavaceae), *Kalanchoë* (Crassulaceae), *Mesembryanthemum* (Aizoaceae) and *Talinum* (Portulacaceae) but no taxa from Orchidaceae. The orchid family, the second largest angiospermous group, including approximately 25,000 species, is a significant lineage of CAM species because approximately 9,000 of its members are estimated to utilize this special pathway^20^. Hence, comprehensive transcriptomic profiling of orchid CAM species should be performed.

Recently, two CAM genomes from orchid species, *Phalaenopsis equetris* and *Dendrobium catenatum* (=*D. officinale*), have been published^9,21,22^, laying a foundation for CAM research. *P. equetris* is an obligate CAM plant^9^ with low metabolic plasticity to environmental changes^23^. However, *D. catenaum* is a facultative CAM plant^24^ that can adjust the intensity of the CAM pathway in response to external stresses, such as drought^25^, which allows researchers to compare varied gradients of physiological activities through manipulating experimental conditions^26^. Consequently, the latter orchid should be considered a better candidate for CAM studies than the former. To date, several RNA-seq datasets for *D. catenatum* have been constructed to examine gene expression in chilling stress^27^, alkaloid biosynthesis^28^, polysaccharides synthesis^29,30^, seed development^31^, and organ-specific regulation^32^ but not in CAM processes.

**Figure 1:**
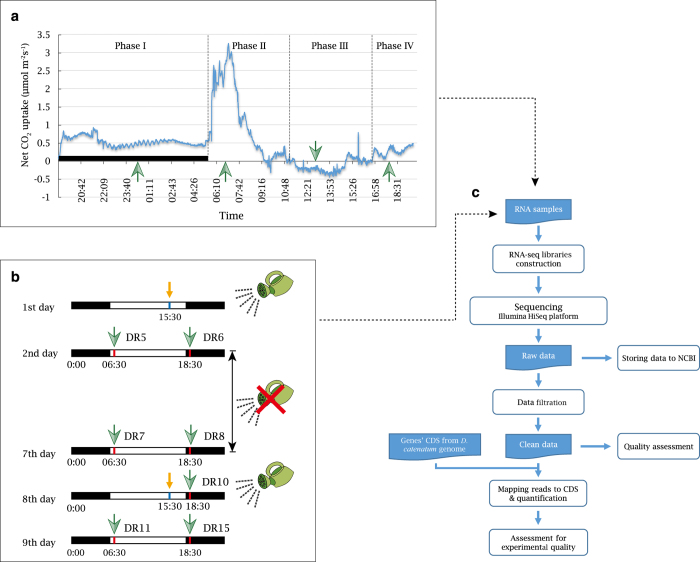
Overview of the experimental design and analysis pipeline. (**a**) The sampling scheme based on typical CAM phases according to the CO_2_ exchange rate during a natural day-night cycle. (**b**) The sampling scheme under sustained drought stress and stress removal. (**c**) Flow chart of the *D. catenatum* RNA-seq experiments and data analyses. Green arrows indicate sample collection times, and yellow arrows indicate watering times. Black bars indicate dark periods.

In this study, we conducted two experiments on *D. catenatum* to generate two RNA-seq datasets. The first experiment aimed to collect the gene expression profile (Dataset I) for CAM and the operation of associated pathways in a diel with four typical phases (Fig. 1a); the second aimed to record the gene expression profile (Dataset II) during the alternation of day and night under drought stress and upon stress removal (Fig. 1b). These experiments yielded 24 samples with a total of 844.4 million reads of transcriptome data from mature leaves (Data Citation 1). Additionally, quality assessments of the data were conducted to verify their reliability (Fig. 1c). We believe that these profiles will help to deepen the comprehensive understanding of the essence of CAM regulation.

## Methods

### Design and sample collection of experiment I

Clones of *D. catenatum* were cultivated in white and transparent pots (5.0 cm in diameter) with sphagnum moss as the matrix. The plants were grown in a greenhouse with temperatures from 22 to 28 °C and relative humidity from 40 to 60%. The experiments were initiated with strong eight-month-old individuals (clones; 12–14 cm height) grown in matrix maintained at approximately 30% volumetric water content, which ensured that these plants did not undergo drought or waterlogging.

During the period when leaf samples were collected, we measured the net CO_2_ exchange rates by Li-6400XT (Li-COR Biosciences Inc., Lincoln, NE, USA) to confirm the four CAM phases^5^ of the plants in a diel. The following parameters were set for the equipment with a double-sided transparent leaf cuvette: photosynthetic photon flux density (PPFD), natural light; cuvette temperature inside, synced with the outside; CO_2_ concentration, atmosphere; flow rate, 200 μmol s^−1^; and cuvette fan speed, fast. According to the curve of CO_2_ exchange rates, leaf samples were collected at 00:30, 06:30, 13:00 and 17:30 (Fig. 1a), which represented Phases I, II, III and IV, respectively. The third and fourth mature leaves from the apex of each individual were harvested and combined as one sample. Three biological replicates were collected for Phase I, five for Phase II, three for Phase III and six for Phase IV. These excised leaves were frozen immediately in liquid nitrogen and stored at −80 °C.

### Design and sample collection of experiment II

Tissue-cultured *D. catenatum* plants from seeds were grown in plastic pots (8.0 cm in diameter) filled with a substrate mix of composted pine bark and small stones. The plants were kept in a growth chamber with a temperature of 28/22 °C (day/night), a photoperiod of 12/12 hr (day/night), a light intensity of ~100 μmol m^−2^s^−1^, a relative humidity of 50/70% (day/night), and watering every two days at 15:30. Vigorous eight-month-old plants with a height of ~12 cm were chosen for the follow-up experiment. Irrigation was performed on the first day, omitted from the second to the seventh day, and recommenced on the eighth day. (Fig. 1b). The mature fourth leaf from the apex of each individual was harvested at both 06:30 and 18:30 (half an hour after light on and off, respectively) on the second, seventh, and ninth days and at 18:30 on the eighth day (Fig. 1b). Each sample time point included only one biological replicate. These samples were frozen immediately in liquid nitrogen and stored at −80 °C.

### RNA extraction, library preparation, and sequencing

Total RNA was isolated from ground tissue using an SDS (prosodium dodecylsulfate) protocol proposed by Cen *et al.*^33^. DNA contamination was removed with recombinant DNase I (Takara Bio, http://www.takarabiomed.com.cn/). When the RNA quality tallied with the standards (see Technical Validation section), RNA-seq libraries were constructed using the TruSeq RNA Sample Prep Kit (Illumina, http://www.illumina.com/). The libraries from experiment I were sequenced in the 150 nt paired-end mode on an Illumina HiSeq2500 platform at Annoroad Gene Technology (Beijing, China; http://www.annoroad.com), and the other libraries were sequenced in the 90 nt paired-end mode on an Illumina Hiseq2000 platform at the Beijing Genomics Institute (Shenzhen, China; http://www.genomics.cn).

### Data filtering and gene quantification

The raw RNA-seq reads were cleaned using the Fastq_clean procedure by Zhang *et al.*^34^, which included trimming adapters and low-quality bases and removing rRNA and viral sequences. The quality control criteria for this filter were set as follows: (1) low-quality bases below phred quality 20 were trimmed from both ends of reads; (2) after the low-quality bases were trimmed, reads containing over two “N” were removed; (3) the reads with length shorter than 75 (for Dataset I) or 50 (for Dataset II) were removed; and (4) BWA^35^ related parameters were set as recommended. The filtering results are listed in Table 1. The clean reads were evaluated using FastQC v0.11.7 (http://www.bioinformatics.babraham.ac.uk/projects/fastqc/), and the assessment results were summarized and visualized using MultiQC v1.3^36^ with the recommended configuration. Salmon ver. 0.9.1^37^ was used to map the clean reads against the primary CDS from the genome (GenBank Assembely ID ASM160598v2) published by Zhang *et al.*^22^ to quantify the gene abundance as read counts. The default settings for Salmon were used. We applied the DESeq2^38^ R package to normalize the read counts.

### Principal component analysis and heatmap illustration

The normalized read count values of each sample in the two datasets were employed in principal component analysis (PCA) with an online tool, three-dimension PCA (http://www.omicshare.com/tools/Home/Soft/seniorpca), using the default parameters. A heatmap for sample clustering of Dataset I was illustration with the R package PoiClaClu^39^.

### Code availability

A R script for read count normalization and heatmap illustration is available in Figshare (Data Citation 2).

## Data Records

The RNA-seq raw data of the two datasets were deposited at the NCBI Sequence Read Archive with BioSample accessions SAMN09267369–SAMN09267385 (Dataset I; Data Citation 1) and SAMN09269105–SAMN09269111 (Dataset II; Data Citation 1). Data Citation 2 contains the R scripts in this study. The files of gene abundance for the two datasets are deposited in Figshare (Data Citation 3). The heatmap for sample clustering of Dataset I is available in Figshare (Data Citation 4).

## Technical Validation

### RNA qualities

The quality of the total RNA was assessed using an Agilent Bioanalyser 2100 (Agilent Technologies). The RNA samples with RNA integrity numbers higher than 7.0 were used to prepare RNA-seq libraries in this study. The RNA quality evaluations are listed in Table 2.

### Quality validation

**Figure 2:**
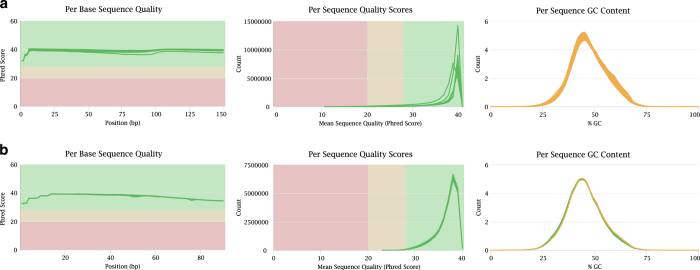
Quality assessment metrics for RNA-seq data. The per base sequence quality (left), per sequence quality scores (middle), and per sequence GC content (right) across all samples of Dataset I (**a**) and Dataset II (**b**).

We applied FastQC to assess the RNA-seq clean data, including the mean per base quality scores, per sequence quality scores, and per sequence GC content. Summary plots are presented in Fig. 2. With respect to both datasets, the quality scores per base were higher than phred quality 30, and almost all sequences had a quality over 20. The GC contents of the samples from both datasets I and II showed a similar normal distribution. Moreover, the RNA-seq data had high mapping rates ranging from 87.13–90.82% (Table 1). These statistics indicated that high-quality RNA-seq reads were obtained for downstream analysis.

**Figure 3:**
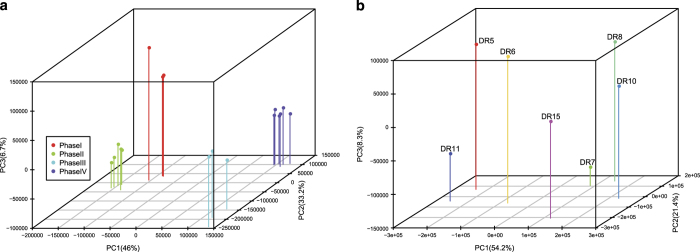
Three-dimensional PCA plots. (**a**) Dataset I and (**b**) Dataset II.

The PCA result (Fig. 3a) showed that the samples in dataset I clustered into four groups corresponding to the four phases in CAM, which was also supported by a sample clustering analysis (Data Citation 4). In Dataset II, the drought stress samples, including DR7, DR8 and DR10, clustered closely, and DR5, DR6 and DR11 were neighbors in the PCA plot (Fig. 3b). The distinctive pattern of samples in the analyses indicated that these transcriptome profiles were valuable for understanding the CAM pathways.

## Additional information

**How to cite this article**: Zou, L.-H. *et al.* RNA-seq transcriptomic profiling of crassulacean acid metabolism pathway in *Dendrobium catenatum. Sci. Data*. 5:180252 doi: 10.1038/sdata.2018.252 (2018).

**Publisher’s note**: Springer Nature remains neutral with regard to jurisdictional claims in published maps and institutional affiliations.

## Supplementary Material



## Figures and Tables

**Table 1:** Statistics of sequencing data for each sample.

Sample	Data set	Sequencing strategy	Raw reads number	Clean reads number	Clean data rate (%)	Mapping rate (%)	Accession
**PI1**	Dataset I	PE150	27660160	27542266	99.57	88.79	SRR7221702
**PI2**	Dataset I	PE150	28518120	28393620	99.56	87.88	SRR7221703
**PI3**	Dataset I	PE150	52075438	51809058	99.49	87.13	SRR7221704
**PII1**	Dataset I	PE150	27285848	27121968	99.40	88.13	SRR7221705
**PII2**	Dataset I	PE150	28178012	28029702	99.47	90.82	SRR7221698
**PII3**	Dataset I	PE150	27530290	27410740	99.57	90.12	SRR7221699
**PII4**	Dataset I	PE150	27772530	27659912	99.59	89.32	SRR7221700
**PII5**	Dataset I	PE150	27628334	27437044	99.31	89.92	SRR7221701
**PIII1**	Dataset I	PE150	27242838	27144468	99.64	87.77	SRR7221696
**PIII2**	Dataset I	PE150	27002996	26922920	99.70	88.66	SRR7221697
**PIII3**	Dataset I	PE150	27902586	27794040	99.61	88.45	SRR7221709
**PIV1**	Dataset I	PE150	28311682	28225282	99.69	88.86	SRR7221710
**PIV2**	Dataset I	PE150	28278086	28182784	99.66	88.69	SRR7221711
**PIV3**	Dataset I	PE150	27896820	27815090	99.71	88.47	SRR7221712
**PIV4**	Dataset I	PE150	27353690	27278754	99.73	88.98	SRR7221713
**PIV5**	Dataset I	PE150	27885292	27775884	99.61	89.76	SRR7221714
**PIV6**	Dataset I	PE150	28472368	28360984	99.61	89.52	SRR7221715
**DR5**	Dataset II	PE90	48084156	47703558	99.21	87.50	SRR7223299
**DR6**	Dataset II	PE90	50314908	49812640	99.00	89.40	SRR7223298
**DR7**	Dataset II	PE90	50331572	49773744	98.89	89.89	SRR7223301
**DR8**	Dataset II	PE90	50258420	49857612	99.20	89.73	SRR7223300
**DR10**	Dataset II	PE90	47991832	47402820	98.77	89.03	SRR7223296
**DR11**	Dataset II	PE90	50357650	49847644	98.99	89.57	SRR7223295
**DR15**	Dataset II	PE90	50046760	49104418	98.12	87.68	SRR7223297
Clean data rate = Clean reads number/Raw reads number. Mapping rates were calculated from the Salmon procedure.							

**Table 2:** RNA sample quality in this study.

Sample	RIN	28 S/18 S	OD260/280	OD260/230
**PI1**	7.4	1.6	2.1	2.1
**PI2**	8.6	1.5	2.4	2.1
**PI3**	7.5	2.1	2.1	2.4
**PII1**	7.0	1.5	2.1	2.3
**PII2**	8.7	1.7	2.4	2.1
**PII3**	8.6	1.9	2.4	2.1
**PII4**	8.8	2.0	2.3	2.1
**PII5**	8.3	1.6	2.4	2.2
**PIII1**	7.1	1.7	2.2	2.3
**PIII2**	8.4	1.5	2.4	2.1
**PIII3**	8.4	1.7	2.4	2.1
**PIV1**	7.9	1.7	2.3	2.1
**PIV2**	7.8	1.6	2.2	2.1
**PIV3**	8.2	1.9	2.2	2.1
**PIV4**	8.1	1.8	2.4	2.1
**PIV5**	7.9	1.9	2.2	2.1
**PIV6**	8.8	1.6	2.4	2.1
**R5**	8.9	2.4	2.2	2.3
**R6**	7.0	2.0	2.1	2.0
**R7**	8.4	1.9	2.1	2.1
**R8**	7.9	1.8	1.9	1.9
**R10**	8.0	2.1	2.0	2.1
**R11**	8.4	2.3	2.2	2.3
**R15**	8.5	1.8	2.0	2.1
